# Long Term Metabolic and Inflammatory Effects of Second-Generation Antipsychotics: A Study in Mentally Disordered Offenders

**DOI:** 10.3390/jpm11111189

**Published:** 2021-11-12

**Authors:** Emilia Vassilopoulou, Dimitris Efthymiou, Evangelia Papatriantafyllou, Maria Markopoulou, Efthymia-Maria Sakellariou, Alina Codruta Popescu

**Affiliations:** 1 Department of Nutritional Sciences and Dietetics, International Hellenic University, 57400 Thessaloniki, Greece; 2 Department of Psychiatry, Division of Neurosciences, School of Medicine, Aristotle University of Thessaloniki, 54124 Thessaloniki, Greece; dimitrisefthy@gmail.com; 3 Department of Forensic Psychiatry, Psychiatric Hospital of Thessaloniki, 56429 Thessaloniki, Greece; marmark33@yahoo.gr; 4 Psychiatric Clinic, General University Hospital, 41110 Larissa, Greece; efthymiamaria@yahoo.com; 5 Department of Abilities Human Sciences, Iuliu Hatieganu University of Medicine and Pharmacy, 400012 Cluj-Napoca, Romania; cpopescu@umfcluj.ro

**Keywords:** forensic psychiatry, antipsychotic drugs, metabolic disturbances

## Abstract

Mentally disordered offenders provided with forensic psychiatric care are often treated with second generation antipsychotic (SGA) medication and experience metabolic and inflammatory side effects. Aim: In this paper, we monitored the three-year fluctuation of selected anthropometric, biochemical, and inflammatory indices in forensic psychiatric patients receiving antipsychotic (AP) medication for more than five years, according to the type of AP. Methods: Thirty-five patients with psychotic disorders were classified into two groups based on the type of AP. Specifically: AP1, related to a lower risk, and AP2, related to an increased risk of weight gain (WG) and metabolic complications. Biochemical, hematological, anthropometric, blood pressure (BP), and medication data were retrieved from the individual medical files. Statistical analysis was performed with SPSS 23. Results: No significant differences in weight and glucose and cholesterol levels were observed, but patients taking AP2 more often needed drugs to control diabetes mellitus (DM), lipidemia, and cardiovascular disease (CVD). In those taking AP1, the mean HDL level decreased significantly over time (*p* < 0.05) and a higher proportion developed higher BP (52.9% of AP1 vs. 16.7% AP2). In the AP2 group the median level of C-reactive protein (CRP) (*p* < 0.001) and the white blood cell count (WBC) increased over the three years (*p* < 0.001). Conclusions: The proposed sub-classification of SGAs into AP1 and AP2, depending on their potential for metabolic and inflammatory effects, might facilitate study of their long-term side-effects but also help in personalized prevention or treatment measures to counteract these side-effects.

## 1. Introduction

### 1.1. Forensic Psychiatry

Forensic psychiatry is a subspecialty of psychiatry, combining forensic medicine and psychiatry [1]. Article 34 of the Greek Penal Code states that a person is not guilty of the act committed if “at the time of the act he was suffering from a mental disorder or a disturbance of consciousness so that he could not understand that his act was wrong, or even if he could understand it, he would not be able to act according to his perception of injustice”. Specifically, the Greek law states that “because he was suffering from a morbid mental dysfunction or disturbance of consciousness, he was not able to fully comprehend that his act was wrong, or even if he could comprehend it, he was not fully capable” [2]. Mentally disordered offenders are provided with forensic psychiatric care, and their treatment often includes the administration of antipsychotic (AP) medication.

### 1.2. Antipsychotic Drugs and Metabolic Disturbances

APs are administered as a first-line treatment for a wide range of psychiatric disorders [3]. They are often used as monotherapy, but also in combination with mood stabilizers or antidepressants, for bipolar disorder, and for major depression, respectively [3].

Although they are considered the cornerstone of treatment for many psychiatric disorders, APs produce metabolic and inflammatory side effects, and their use is associated with weight gain (WG) and the development of obesity, metabolic syndrome, dyslipidemia, type II diabetes mellitus (DM), hypertension, and cardiovascular disease (CVD) [4,5,6], which increase morbidity and mortality in psychiatric patients [3,7,8].

Treatment with APs is closely linked to the basic components of metabolic syndrome (i.e., WG, glucose intolerance and dyslipidemia) and, to a lesser extent, hypertension and CVD [7,9,10]. Second-generation APs (SGAs), such as clozapine and olanzapine, cause greater WG, while quetiapine, risperidone, paliperidone, and iloperidone have an intermediate risk, and aripiprazole, amisulpride, ziprasidone, asenapine, and lurasidone have little or no effect on body weight [7]. WG is greater in drug naïve patients [7,11], and the side effects, therefore, appear to depend on the degree of previous exposure to Aps [7,11]. WG associated with APs is more rapid in the first few weeks of treatment, gradually slowing, and often stabilizing, within the first year [11,12]. Several studies have documented milder WG and cardiometabolic complications, depending on the type of AP, and specifically with aripiprazole, amisulpride, quetiapine XR, paliperidone, and ziprasidone. Complications are reported to be more severe in treatment with olanzapine, asenapine, clozapine and risperidone [13,14,15]. Multipharmacotherapy, commonly used for forensic psychiatric patients, has been associated with greater WG than monotherapy [16].

The risk of developing metabolic syndrome is greater with clozapine, olanzapine, and chloropromazine, moderate with quetiapine, risperidone, paliperidone, amisulpride and serendon, and generally low with aripiprazole and ziprasidone [7]. SGAs can be classified according to their risk of cardiovascular and metabolic adverse reactions, as follows: clozapine = olanzapine > quetiapine ≥ risperidone = paliperidone > amisulpride ≥ ripiprazole ≥ ziprasidone.

Considering the variable side effects of the APs, we hypothesized that we can sub-group them according to the range of the WG they provoke, according to the relevant literature, into two categories, the first including the APs causing milder WG, and the second causing more severe WG. The aim of the current study was to monitor the three-year fluctuation of selected anthropometric, metabolic and inflammatory factors in forensic psychiatric patients who had been receiving APs for more than five years, according to the type of AP.

## 2. Materials and Methods

### 2.1. Patients

In this prospective, 3-year study, we included 35 patients with psychotic disorders, who were all offenders, classified as irresponsible for their actions and hospitalized in the Thessaloniki Psychiatric Hospital after a relevant judicial decision. Classification of the mental disorders was made according to the ICD-10 classification by qualified psychiatrists at the hospital.

The 35 patients had all been receiving SGAs for at least 5 years at the start of the study (T1). The patients were classified into two groups, based on the type of SGA. Specifically: AP1, associated with lower to moderate risk of WG from the baseline weight and less risk of metabolic complications (namely aripiprazole, amisulpride, quetiapine, risperidone and ziprasidone), and AP2, associated with a higher risk of WG from the baseline weight and greater risk of metabolic complications (namely paliperidone, olanzapine, asenapine, and clozapine) [13,14,15]. Clinical data were retrieved from the hospital records of the patients, including the SGAs used, changes in prescribed SGAs over time, and medications used for the treatment of other chronic disease, such as lipidemia, CVD, and type II DM.

The study was approved by the scientific committee of the hospital (code number: Δ3B/34800/21/07/2020) and the research Ethics Committee of the Aristotle University of Thessaloniki (code number 4/26.01.2021) and complied with the International Code of Medical Ethics of the World Medical Association and the Helsinki Declaration.

### 2.2. Biochemical/ Hematological Assessment

Venous blood samples were collected after overnight fasting from all participants every first quarter of the years in the three-year study period (T1: 2018, T2: 2019, T3: 2020), as part of their routine monitoring procedure. Serum and plasma aliquots were isolated and stored at −80 °C until further analysis.

The erythrocyte sedimentation rate (ESR),white blood cell count (WBC), platelet count (PLT) and concentrations of blood glucose (GL), serum total cholesterol, triglycerides (TG), high density lipoprotein cholesterol (HDL), low density lipoprotein cholesterol (LDL), and high-sensitivity C-reactive protein (CRP), vitamin B12 and folic acid were measured by automatic analyzer (Toshiba TBA 120FR; Toshiba Medical Systems Co., Ltd., Tokyo, Japan) under standard conditions, on the hospital premises.

### 2.3. Anthropometric Measurements

Anthropometric measurements were made on all participants on the morning of the blood collection, after fasting for at least 8 h, by one trained investigator. Height was measured to the nearest 0.1 cm, using a commercial stadiometer (Leicester Height Measure, Invicta Plastics Ltd., Oadby, UK) with the participants barefoot, their shoulders in a relaxed position, their arms hanging freely, and their heads in the Frankfort horizontal plane. The participants were weighed barefoot and in light clothing to the nearest 0.1 kg, using a TANITA RD-545 (“RD-545-Connected smart scale|Tanita Official Store”, n.d.). Body mass index (BMI) was calculated from the current weight and height (weight (kg) by height squared (m^2^)). The waist circumference (WC) was measured with a SECA flexible, inextensible measuring tape, with an accuracy of 1 mm, on a horizontal plane, after exhalation, at a point equidistant from the lowest floating rib and the upper border of the iliac crest.

### 2.4. Blood Pressure Determination

Arterial blood pressure (BP) was recorded to the nearest 2 mmHg, using a mercury sphygmomanometer with the arm supported at heart level, after the subject had been sitting quietly for 10 min. One trained member of the research team took three separate readings at 1-min intervals. The average of the last two readings was used for analysis.

### 2.5. Statistical Analysis

The statistical package SPSS 23 was used for analysis of the data. The values were expressed as mean ± standard deviation (SD), or median (interquartile range (IQR)). Where the possible difference between the biochemical characteristics at two time periods was checked, the *t*-test for 2 dependent samples or the Wilcoxon test was used, depending on the regularity of the differences. Significant explore the differences between the groups of patients based on the psychotic drug they were receiving, AP1 or AP2, the *t*-test or the non-parametric Mann-Whitney test was applied, depending on the normality of the data. The Pearson chi-squared independence test was used to test for a significant relationship between quality variables. In the case of values less than 5 in more than 20% of the cells in the correlation table, the chi-squared test was replaced by the Monte Carlo method.

## 3. Results

The study sample of 35 forensic psychiatric patients included 31 males and 4 females, of whom 17 were receiving AP1 and 18 AP2. Their general characteristics are presented in Table 1. The age range in the two subgroups was similar; the mean age of patients taking AP1 was 52.35 ± 12.68 years and of those taking AP2 48.56 ± 12.65 years (*p* = 0.38). All the patients received the same SGA over the study period. The patients receiving AP2 medication more frequently needed to take anti-diabetic, anti-lipidemic and medications for CVD (i.e., heart rate lowering agents, such as ivabradine; beta blockers, such as propranolol; and calcium channel blockers, such as amlodipine), in comparison to patients receiving AP1.

The anthropometric and biochemical measurements at the three yearly time points (T1, T2, T3) are presented in Table 2 (A,B,C).

Table 3 shows the parameters at the three yearly time points, according to the antipsychotic medication AP1 and AP2 being taken by the patients. No significant changes in weight, BMI, GL, and cholesterol were observed, either in the same group or between the subgroups over the three time periods (Table 3).

**Figure 1:**
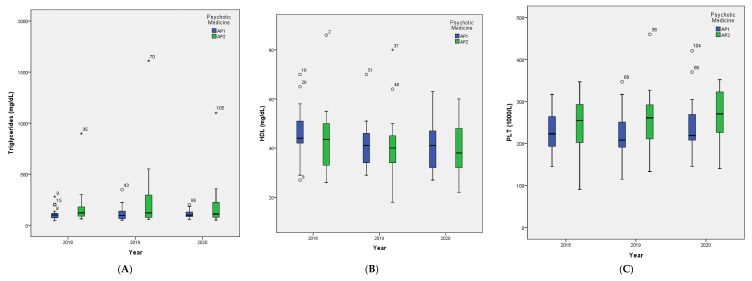

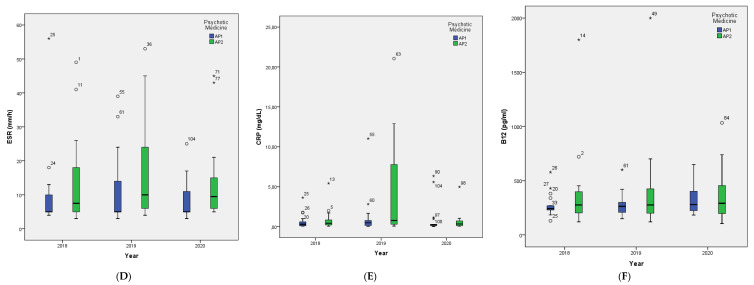
Box plots illustrating the changes of blood parameters in the subgroups of psychotic patients, depending on the antipsychotic medication (AP1, AP2), at the three yearly time points, 2018 (T1), 2019 (T2), and 2020 (T3). (**A**) triglycerides; (**B**) high density lipoprotein (HDL); (**C**) platelet count (PLT); (**D**) erythrocyte sedimentation rate (ESR); (**E**) C-reactive protein (CRP); (**F**) vitamin B12. AP1, antipsychotics related to a low to moderate risk of WG, namely aripiprazole, amisulpride, quetiapine, risperidone, and ziprasidone; AP2, antipsychotics related to a higher risk of WG, namely paliperidone, olanzapine, asenapine, clozapine.

Figure 1 shows box plots demonstrating significant differences in blood parameters between the measurements at the three time points, T1 (2018), T2 (2019), and T3 (2020).

In the patients taking AP2, the median level of triglycerides showed a reduction of 11 units between T2 and T3 (*p* = 0.01) (Figure 1A). The mean level of HDL decreased significantly in all patients between T1 (45.03 ± 12.48 mg/dL) and T2 (41.77 ± 12.0148 mg/dL) (*p* < 0.001) (Figure 1B). Specifically, the patients taking AP1 showed a significant reduction in mean level of HDL between T1 (46.47 ± 11.17 mg/dL) and T2 (42.06 ± 9.85 mg/dL) (*p* = 0.018), and between T2 and T3 (40.24 ± 9.69 mg/dL) (*p* = 0.038) (Figure 1C).

PLT increased significantly in all patients between T2 (239.77 ± 69.37103/L) and T3 (251.66 ± 68.74103/L) (*p* = 0.01) (Figure 1D). Similarly, the ESR increased significantly between T2 (7 mm/h, IQR 16 mm/h) and T3 (8 mm/h, IQR 8 mm/h (*p* = 0.01) (Figure 1E). An increase in median CRP level of the total sample was observed between T1 (0.4 mg/L, IQR 0.70 mg/L) and T2 (0.51 mg/L, IQR 2.64 mg/L) (*p* = 0.008), and a significantly lower level at T3 (0.25 mg/L, IQR 0.6 mg/L) (*p* = 0.011) (Figure 1F). In the AP2 group (Figure 1G) the median CRP level increased between T1 (0.43 mg/L, IQR 0.67 mg/L) and T2 (0.78 mg/L, IQR 7.56 mg/L) (*p* = 0.006) and between T2 and T3 (0.34 mg/L, IQR 0.61 mg/L) (*p* = 0.006). Finally, in the patients taking AP1, a significant change in mean B12 level was observed between T2 (278.82 ± 111.03 pg/mL) and T3 (331.82 ± 144.31 pg/mL) (*p* = 0.04) (Figure 1F).

**Figure 2:**
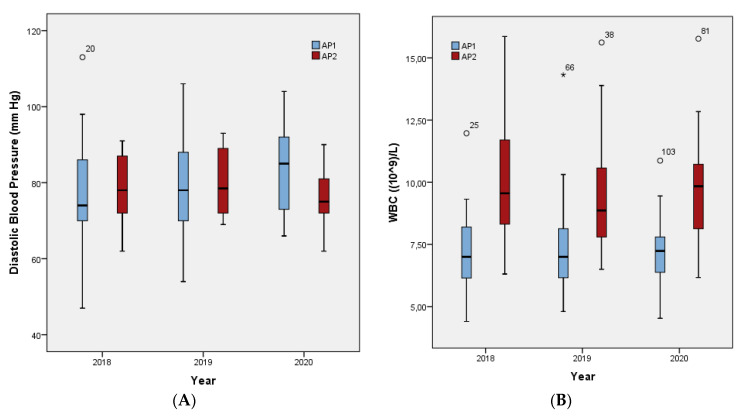
Box plots illustrating parameters that showed a significant difference in forensic psychiatric patients receiving AP1 and AP2, at the three yearly time points, 2018 (T1), 2019 (T2) and 2020 (T3). (**A**) diastolic blood pressure, (**B**) white blood cells (WBC). AP1, antipsychotics related to a low to moderate risk of WG, namely aripiprazole, amisulpride, quetiapine, risperidone, and ziprasidone; AP2, antipsychotics related to a higher risk of WG, namely paliperidone, olanzapine, asenapine, and clozapine.

The differences in measurements between the AP1 and AP2 groups at the different time points (Table 3) were statistically significant, as shown also in Figure 2A,B, for diastolic BP at T3 (83.82 ± 11.32 mm Hg in AP1 vs. 76.72 ± 7.40 mm Hg in AP2, *p* = 0.03), and for WBC, which was higher in patients taking AP2 at all three time points (T1: *p* <0.001, T2: *p* = 0.019, T3: *p* < 0.001).

Table 4 presents the variables being considered for correlation with the development of metabolic syndrome at each observation period, depending on the AP that the patients were receiving, AP1 or AP2. The only metabolic syndrome parameter that appeared to be influenced over time was the diastolic BP. Specifically, 52.9% of patients receiving AP1 developed diastolic BP higher than 85 mm Hg, while for those taking AP2 the rate was 16.7%.

## 4. Discussion

This three-year prospective investigation of forensic psychiatric patients who had been receiving antipsychotic therapy more than five years revealed that, despite the limited changes over the study period in the anthropometric parameters studied (i.e., weight, BMI, and WC), the metabolic and inflammatory effects of the long-term use of APs, as indicated by the need for specific medication, are ongoing.

Specifically, a large proportion of the patients receiving AP2 required diabetic, anti-lipidemic, and medication for CVD to treat metabolic side effects of the SGA, and considerably less than those receiving AP1. The systematic monitoring of these hospitalized forensic psychiatric patients, and the regulation of the relevant cardiovascular and metabolic changes with appropriate medication, can justify the non-significant changes in levels of GL and total cholesterol observed during the study period in the patients, both within the same group and between the two study groups (AP1 and AP2). One exception was the level of triglycerides, which was significantly higher at the T3 than at the T2 period in the patients taking AP2. AP2 have been correlated with direct dysregulation of the metabolism of triglycerides, for which various mechanisms have been proposed, including stimulation of the production and secretion of hepatic triglycerides by inhibiting lipoprotein caused by triglyceride lipase hydrolysis [17,18].

In patients taking AP1, we observed a significant decrease in HDL levels over the three years. Ziprasidone, categorized in the AP1 medications, has been proposed repeatedly as the AP with the most benign metabolic profile [19,20,21]. Quetiapine, also categorized as an AP1, has been reported to cause higher levels of LDL cholesterol [22], a higher ratio of triglyceride to HDL-cholesterol [23], and greater WG [20,24]. Aripripazole, ziprasidone, and quetiapine have also been documented to provoke similar metabolic changes [25]. Patients receiving AP1 need monitoring and dietary and lifestyle intervention in order to prevent reduction in HDL [26].

PLT and ESR showed significant increases between T2 and T3 in all the patients. A significant fluctuation in CRP was observed in all the patients, with an initial increase from T1 to T2 and a significant decrease from T2 to T3. In the patients taking AP2, these changes were statistically significant, indicating the greater effect of these drugs on inflammation. In line with the CRP changes, the patients taking AP2 showed significantly higher WBC at all time periods. Some clinical studies have shown a deterioration in metabolic markers, with increased levels of CRP after the start of SGAs, although some report minimal or no change and others report reduced levels of CRP after starting these drugs. Inconsistent findings between studies can be explained by the heterogeneous patient populations, differences in methodology, and small sample sizes without statistical power [27]. APs have been linked to the development of inflammatory lesions and lymphocytic infiltration in the myocardium [28,29,30,31].

In relation to the differences in the anthropometric indices and other metabolic and inflammatory markers according to the type of AP treatment, the AP2 group had a significantly higher mean diastolic BP following long-term use. WG and obesity, dyslipidemia, insulin resistance, and type 2 DM are documented to be more common in patients treated with Aps [7,25,32]. The metabolic effects, including obesity, dyslipidemia, hyperglycemia and type 2 DM, can increase the risk of CVD [18,33]. Several studies have shown that patients using olanzapine, categorized in this study as AP2, occasionally exhibit hypertriglyceridemia, hypercholesterolemia, and increased serum low density lipoprotein, either with or without WG [34,35,36]. A study of 242 people with severe mental disorders revealed that, despite similar BMI, hypertriglyceridemia was much more widespread in those treated with clozapine or olanzapine than in non-treated healthy patients [18,37,38]. Kang and Lee reported that individuals treated with clozapine and olanzapine showed significant insulin resistance and attenuated glucose efficiency compared with those treated with risperidone. These findings, which are supported by our study, indicate that SGAs directly affect metabolic disorders, rather than simply increasing the effects of known risk factors such as obesity [18]. In patients receiving AP2, appropriate personalized nutritional intervention could play a significant role in preventing or controlling their cardiometabolic and inflammatory complications [39,40].

The metabolic side effects of SGAs have become a subject of major concern for clinicians. Development of DM at the start of clozapine treatment was observed in 36.6% of patients in a five-year period, with the suggestion that olanzapine and clozapine exert the greatest diabetogenic effect [41,42]. It was reported that 16.9% of 260 clozapine users in a Dutch hospital had DM [41], and a study in Sweden found that in 60 people who were receiving clozapine, 12% had type 2 DM [42]. The results of the study of Vázquez-Bourgon and colleagues [25] provide further indications of the metabolic effects of long-term treatment with AP1 (aripiprazole, ziprasidone and quetiapine). After one year of treatment, these drugs provoked similar increases in weight and BMI, and in blood levels of GL, cholesterol and triglycerides, suggesting that none of these medications can be considered metabolically neutral [25].

Finally, the decrease over the three years of the study in the B12 level in the patient staking AP1 was significant. B12 deficiency has been linked with the pathophysiology of psychosis; it leads to a rise in homocysteine and cortisol, contributing to neuropsychiatric complications such as mental confusion, memory changes, mood disorder, violent behavior, delirium, and paranoid psychosis [43]. Maintaining B12 at normal levels, through diet or supplements, is suggested for the regulation of psychotic symptoms and for the reduction of the risk of coronary disease and insulin resistance [44].

In our study, the small sample size does not permit us to generalize our results and proceed with certainty to conclusions on the effects of the individual APs or the subgroups, but the findings warrant further investigation. All of the patients in this study had been receiving SGAs for more than five years, but there was a lack of information on the exact time of drug initiation. In this study, due to the small sample size, the SGAs were subgrouped into two categories, with those provoking mild and moderate complications being combined in the same group. The study population in both the groups was overweight (mean BMI > 25) at all time points, and therefore the risk of metabolic complications, such as CVD and DM, was high in both. Lacking the information about their weight at the initiation of SGAs and the duration of exposure to specific drugs before the start of the study, we cannot assume that the metabolic and inflammatory side effects measured in the study period are related only relevant to the current medication use.

## 5. Conclusions

We have proposed here the use of a sub-classification of SGAs into AP1 and AP2, depending on their potential for metabolic and inflammatory effects, to facilitate the study of relevant long-term side-effects and their management. In our study, this classification revealed that although all the forensic psychiatric study patients were overweight, those receiving AP2 medication more often needed other drugs to control DM, lipidemia and CVD, while deleterious effects on levels of HDL and B12 were observed in the patients taking AP1. Despite the study limitations, we believe that the findings justify further investigation into the long-term effects of the APs, under the proposed classification, as it can facilitate, in the clinical setting, the development of personalized prevention or treatment measures according to the relevant metabolic and inflammatory side-effects, specifically, lifestyle-based intervention in those taking AP1 and dietary intervention in those taking AP2.

## Figures and Tables

**Table 1:** Demographic and clinical characteristics and medication of the study population of forensic psychiatric patients (N = 35).

		Ν	%
Gender	Females	4	11.4%
	Males	31	88.6%
Antipsychotic medication	AP1	17	48.6%
	AP2	18	51.4%
Diagnosis	Schizophrenia	24	68.6%
	Other non-organic psychosis	7	20%
	Chronic delusional disorders	1	2.95
	Mixed and other personality disorders	1	2.9%
	Mental disorders and behavioral disorders	2	5.7%
Other medication	Anticoagulants (1st year)	2	5.7%
	Anticoagulants (2ndyear)	5	14.3%
	Anticoagulants (3rd year)	5	14.3%
	Antihypertensives (1st year)	4	11.4%
	Antihypertensives (2ndyear)	7	20%
	Antihypertensives (3rdyear)	8	22.9%
	Antidiabetic (1st year)	4	11.4%
	Antidiabetic (2nd year)	5	14.3%
	Antidiabetic (3rd year)	4	11.4%
	Antilipidemics (1st year)	5	14.3%
	Antilipidemics(2nd year)	7	20%
	Antilipidemics (3rd year)	9	25.7%
	Medication for CVD (1st year)	10	28.6%
	Medication for CVD (2ndyear)	10	28.6%
	Medication for CVD (3rd year)	11	31.4%

N, number of patients; %, percentage of patients; CVD, cardiovascular disease; AP1, antipsychotics related to a low to moderate risk of WG, namely aripiprazole, amisulpride, quetiapine, risperidone, and ziprasidone; AP2, antipsychotics related to a higher risk of WG, namely paliperidone, olanzapine, asenapine, clozapine.

**Table 2:** (**A**). In-group difference tests on the overall sample and the subgroups of forensic psychiatric patients receiving antipsychotic medication (AP1 and AP2) in the years 2018 (T1) and 2019 (T2). (**B**). In-group difference tests on the overall sample and the subgroups of forensic psychiatric patients receiving antipsychotic medication (AP1 and AP2) in the years 2019 (T2) and 2020 (T3). (**C**). In-group difference tests on the overall sample and the subgroups of forensic psychiatric patients receiving antipsychotic medication (AP1 and AP2) in the years 2018 (T1) and 2020 (T3).

**(A)**									
Variables	Total T1	Total T2	*p*-Value	AP1 T1	AP1 T2	*p*-Value	AP2 T1	AP2 T2	*p*-Value
Weight (kg)	85.26 (15.59) ^1^	86.37 (16.40) ^1^	0.38 ^2^	86.47 (18.54) ^1^	88.82 (18.74) ^1^	0.17 ^2^	84.11 (12.64) ^1^	84.06 (14.00) ^1^	0.98 ^2^
BMI (kg/m^2^)	29.09 (6.05) ^1^	29.42 (5.88) ^1^	0.26	30.10 (7.09) ^1^	30.84 (6.59) ^1^	0.21 ^2^	28.14 (4.88) ^1^	28.07 (4.95) ^1^	0.61
Glucose (mg/dL)	94.00 (13.00)	94.00 (24.00)	0.19	94.00 (12.00)	94.00 (26.00)	0.08	97.17 (11.32)	94.50 (27.00)	0.65
Cholesterol (mg/dL)	174.71 (36.18) ^1^	171.11 (38.81) ^1^	0.69	166.00 (31.77) ^1^	164.65 (43.55) ^1^	0.30	182.94 (38.99) ^1^	177.22 (33.86) ^1^	0.65
Triglyce-rides (mg/dL)	109.00 (69.00)	108.00 (126.00)	0.12	100.00 (58.00)	119.82 (76.89) ^1^	0.69 ^2^	122.00 (110.00)	121.50 (228.00)	0.07
HDL (mg/dL)	45.03 (12.48) ^1^	41.77 (12.01) ^1^	0.00	46.47 (11.17)	42.06 (9.83)	0.01	43.67 (13.78)	41.50 (14.06)	0.107 ^2^
LDL (mm Hg)	97.23 (32.24) ^1^	98.91 (31.76) ^1^	0.46	94.06 (28.17) ^1^	99.12 (37.69) ^1^	0.98	100.22 (36.22) ^1^	98.72 (26.07) ^1^	0.28
Systolic BP (mm Hg)	121.23 (12.93) ^1^	123.94 (14.85) ^1^	0.13 ^2^	120.71 (15.34) ^1^	125.12 (17.00) ^1^	0.17 ^2^	121.72 (10.59) ^1^	122.83 (12.89) ^1^	0.52 ^2^
Diastolic BP (mm Hg)	78.26 (11.91) ^1^	79.63 (11.67) ^1^	0.39	78.00 (15.17) ^1^	80.06 (14.76) ^1^	0.46 ^2^	78.50 (8.18) ^1^	79.22 (8.17) ^1^	0.81
WBC (10^3^/L)	8.75 (2.57) ^1^	8.65 (2.58) ^1^	0.91	7.35 (1.91) ^1^	7.62 (2.29) ^1^	0.33	9.56 (3.47)	9.62 (2.51) ^1^	0.41
PLT (10^3^/L)	240.51 (61.17) ^1^	239.77 (69.37) ^1^	0.94 ^2^	227.82 (54.31) ^1^	222.35 (60.97) ^1^	0.55 ^2^	252.50 (66.30) ^1^	256.22 (74.41) ^1^	0.82 ^2^
ESR (mm/h)	5.00 (7.00)	7.00 (16.00)	0.11	5.00 (5.50)	5.00 (11.00)	0.68	7.50 (13.50)	10.00 (21.80)	0.06
CRP (mg/dL)	0.40 (0.70)	0.51 (2.64)	0.01	0.30 (0.70)	0.48 (0.76)	0.33	0.43 (0.67)	0.78 (7.56)	0.01
B12 (pg/mL)	250.00 (167.00)	270.00 (181.00)	0.13	243.00 (95.00)	278.82 (111.03) ^1^	0.32 ^2^	276.00 (206.00)	276.00 (229.00)	0.25
Folicacid (ng/mL)	4.00 (3.10)	3.90 (2.80)	0.79	4.40 (3.20)	4.00 (4.70)	0.87	3.95 (1.80)	3.70 (2.10)	0.47
**(B)**									
Variables	Total T2	Total T3	*p*-Value	AP1 T2	AP1 T3	*p*-Value	AP2 T2	AP2 T3	*p*-Value
Weight (kg)	86.37 (16.40) ^1^	85.34 (16.41) ^1^	0.19	88.82 (18.74) ^1^	86.53 (19.34) ^1^	0.10	84.06 (14.00) ^1^	84.22 (13.57) ^1^	0.93 ^2^
BMI (kg/m^2^)	29.42 (5.88) ^1^	29.04 (5.75) ^1^	0.20	30.84 (6.59) ^1^	30.01 (6.79) ^1^	0.12	28.07 (4.95) ^1^	28.11 (4.56) ^1^	0.95 ^2^
Glucose (mg/dL)	94.00 (24.00)	95.00 (16.00)	0.30	94.00 (26.00)	95.00 (15.00)	0.33	94.50 (27.00)	94.50 (22.00)	0.47
Cholesterol (mg/dL)	171.11 (38.81) ^1^	164.34 (35.30) ^1^	0.75	164.65 (43.55) ^1^	159.18 (29.52) ^1^	0.86	177.22 (33.86) ^1^	160.50 (60.00)	0.57
Triglyce-rides (mg/dL)	108.00 (126.00)	153.46 (105.00)	0.17	119.82 (76.89) ^1^	108.00 (40.95) ^1^	0.55	121.50 (228.00)	110.50 (151.00)	0.01
HDL (mg/dL)	41.77 (12.01) ^1^	39.71 (10.48) ^1^	0.084 ^2^	42.06 (9.83)	40.24 (9.69)	0.03	41.50 (14.06)	39.22 (11.43)	0.21 ^2^
LDL (mm Hg)	98.91 (31.76) ^1^	95.29 (30.57) ^1^	0.90	99.12 (37.69) ^1^	96.12 (22.07) ^1^	0.51	98.72 (26.07) ^1^	86.50 (59.00)	0.51
Systolic BP (mm Hg)	123.94 (14.85) ^1^	126.86 (21.12) ^1^	0.44	125.12 (17.00) ^1^	128.00 (23.00)	0.64	122.83 (12.89) ^1^	119.50 (25.00)	0.70 ^2^
Diastolic BP (mm Hg)	79.63 (11.67) ^1^	79.00 (16.00)	0.44	80.06 (14.76) ^1^	83.82 (11.32) ^1^	0.66	79.22 (8.17) ^1^	76.72 (7.40) ^1^	0.09 ^2^
WBC (10^3^/L)	8.65 (2.58) ^1^	8.57 (2.40) ^1^	0.86 ^2^	7.62 (2.29) ^1^	7.16 (1.60) ^1^	0.87	9.62 (2.51) ^1^	9.91 (2.29) ^1^	0.63
PLT (10^3^/L)	239.77 (69.37) ^1^	251.66 (68.74) ^1^	0.01	222.35 (60.97) ^1^	238.88 (74.27) ^1^	0.07 ^2^	256.22 (74.41) ^1^	263.72 (62.78) ^1^	0.07
ESR (mm/h)	7.00 (16.00)	8.00 (8.00)	0.05	5.00 (11.00)	5.00 (6.00)	0.20	10.00 (21.80)	9.50 (10.50)	0.13
CRP (mg/dL)	0.51 (2.64)	0.25 (0.60)	<0.01	0.48 (0.76)	0.21 (0.51)	0.34	0.78 (7.56)	0.34 (0.61)	<0.01
B12 (pg/mL)	270.00 (181.00)	290.00 (225.00)	0.10	278.82 (111.03) ^1^	331.82 (144.31) ^1^	0.04	276.00 (229.00)	359.39 (232.18) ^1^	0.73
Folicacid (ng/mL)	3.90 (2.80)	4.10 (9.10)	0.16	4.00 (4.70)	3.80 (13.20)	0.30	3.70 (2.10)	4.20 (5.40)	0.44
**(C)**									
Variables	Total T1	Total T3	*p*-Value	AP1 T1	AP1 T3	*p*-Value	AP2 T1	AP2 T3	*p*-Value
Weight (kg)	85.26 (15.59) ^1^	85.34 (16.41) ^1^	0.84	86.47 (18.54) ^1^	86.53 (19.34) ^1^	0.98 ^2^	84.11 (12.64) ^1^	84.22 (13.57) ^1^	0.95 ^2^
BMI (kg/m^2^)	29.09 (6.05) ^1^	29.04 (5.75) ^1^	0.82	30.10 (7.09) ^1^	30.01 (6.79) ^1^	0.93 ^2^	28.14 (4.88) ^1^	28.11 (4.56) ^1^	0.96 ^2^
Glucose (mg/dL)	94.00 (13.00)	95.00 (16.00)	0.31	94.00 (12.00)	95.00 (15.00)	0.31	97.17 (11.32)	94.50 (22.00)	0.67
Cholesterol (mg/dL)	174.71 (36.18) ^1^	164.34 (35.30) ^1^	0.16 ^2^	166.00 (31.77) ^1^	159.18 (29.52) ^1^	0.31	182.94 (38.99) ^1^	160.50 (60.00)	0.31
Triglyce-rides (mg/dL)	109.00 (69.00)	153.46 (105.00)	0.59	100.00 (58.00)	108.00 (40.95) ^1^	1.00	122.00 (110.00)	110.50 (151.00)	0.57
HDL (mg/dL)	45.03 (12.48) ^1^	39.71 (10.48) ^1^	<0.01 ^2^	46.47 (11.17)	40.24 (9.69)	<0.01 ^2^	43.67 (13.78)	39.22 (11.43)	0.08 ^2^
LDL (mm Hg)	97.23 (32.24) ^1^	95.29 (30.57) ^1^	0.73 ^2^	94.06 (28.17) ^1^	96.12 (22.07) ^1^	0.73 ^2^	100.22 (36.22) ^1^	86.50 (59.00)	0.56 ^2^
Systolic BP (mm Hg)	121.23 (12.93) ^1^	126.86 (21.12) ^1^	0.08	120.71 (15.34) ^1^	128.00 (23.00)	0.09	121.72 (10.59) ^1^	119.50 (25.00)	0.33 ^2^
Diastolic BP (mm Hg)	78.26 (11.91) ^1^	79.00 (16.00)	0.40	78.00 (15.17) ^1^	83.82 (11.32) ^1^	0.10 ^2^	78.50 (8.18) ^1^	76.72 (7.40) ^1^	0.13 ^2^
WBC (10^3^/L)	8.75 (2.57) ^1^	8.57 (2.40) ^1^	0.66 ^2^	7.35 (1.91) ^1^	7.16 (1.60) ^1^	0.72 ^2^	9.56 (3.47)	9.91 (2.29) ^1^	0.80 ^2^
PLT (10^3^/L)	240.51 (61.17) ^1^	251.66 (68.74) ^1^	0.11 ^2^	227.82 (54.31) ^1^	238.88 (74.27) ^1^	0.29 ^2^	252.50 (66.30) ^1^	263.72 (62.78) ^1^	0.25 ^2^
ESR (mm/h)	5.00 (7.00)	8.00 (8.00)	0.45	5.00 (5.50)	5.00 (6.00)	0.61	7.50 (13.50)	9.50 (10.50)	0.15
CRP (mg/dL)	0.40 (0.70)	0.25 (0.60)	0.46	0.30 (0.70)	0.21 (0.51)	0.96	0.43 (0.67)	0.34 (0.61)	0.31
B12 (pg/mL)	250.00 (167.00)	290.00 (225.00)	0.05 ^2^	243.00 (95.00)	331.82 (144.31) ^1^	0.04	276.00 (206.00)	359.39 (232.18) ^1^	0.57
Folicacid (ng/mL)	4.00 (3.10)	4.10 (9.10)	0.11	4.40 (3.20)	3.80 (13.20)	0.09	3.95 (1.80)	4.20 (5.40)	0.51

^1^ Mean value and standard deviation are calculated for regularly distributed variables. In non-regularly distributed median and interquartile range were calculated. ^2^ *t*-test was applied for two dependent samples. All other tests were performed with the non-parametric Wilcoxon test. AP1, antipsychotics related to a low to moderate risk of WG, namely aripiprazole, amisulpride, quetiapine, risperidone, and ziprasidone; AP2, antipsychotics related to a higher risk of WG, namely paliperidone, olanzapine, asenapine, clozapine. BMI, body mass index; BP, arterial blood pressure; WBC, white blood cell count; PLT, platelets: ESR, erythrocyte sedimentation rate; CRP, C-reactive protein.

**Table 3:** Differences between subgroups of forensic psychiatric patients receiving antipsychotic medication AP1 and AP2 at the three yearly time points (T1: 2018, T2: 2019, T3: 2020).

Variable	T1			T2			T3		
	AP1	AP2	*p*-Value	AP1	AP2	*p*-Value	AP1	AP2	*p*-Value
Weight (kg)	86.47 (18.54) ^1^	84.11 (12.64) ^1^	0.66 ^2^	88.82 (18.74) ^1^	84.06 (14.00) ^1^	0.39 ^2^	86.53 (19.34) ^1^	84.22 (13.57) ^1^	0.68 ^2^
BMI (kg/m^2^)	30.10 (7.09) ^1^	28.14 (4.88) ^1^	0.34 ^2^	30.84 (6.59) ^1^	28.07 (4.95) ^1^	0.16 ^2^	30.01 (6.79) ^1^	28.11 (4.56) ^1^	0.33 ^2^
Glucose (mg/dL)	94.00 (12.00)	97.17 (11.32)	0.80	94.00 (26.00)	94.50 (27.00)	0.98	95.00 (15.00)	94.50 (22.00)	0.76
Cholesterol (mg/dL)	166.00 (31.77) ^1^	182.94 (38.99) ^1^	0.17 ^2^	164.65 (43.55) ^1^	177.22 (33.86) ^1^	0.34 ^2^	159.18 (29.52) ^1^	160.50 (60.00)	0.96
Triglycerides (mg/dL)	100.00 (58.00)	122.00 (110.00)	0.11	119.82 (76.89) ^1^	121.50 (228.00)	0.11	108.00 (40.95) ^1^	110.50 (151.00)	0.32
HDL (mg/dL)	46.47 (11.17)	43.67 (13.78)	0.51 ^2^	42.06 (9.83)	41.50 (14.06)	0.89 ^2^	40.24 (9.69)	39.22 (11.43)	0.78 ^2^
LDL (mm Hg)	94.06 (28.17) ^1^	100.22 (36.22) ^1^	0.58	99.12 (37.69) ^1^	98.72 (26.07) ^1^	0.97	96.12 (22.07) ^1^	86.50 (59.00)	0.64
Systolic BP (mm Hg)	120.71 (15.34) ^1^	121.72 (10.59) ^1^	0.82 ^2^	125.12 (17.00) ^1^	122.83 (12.89) ^1^	0.65 ^2^	128.00 (23.00)	119.50 (25.00)	0.64
Diastolic BP (mm Hg)	78.00 (15.17) ^1^	78.50 (8.18) ^1^	0.90 ^2^	80.06 (14.76) ^1^	79.22 (8.17) ^1^	0.84 ^2^	83.82 (11.32) ^1^	76.72 (7.40) ^1^	0.03 ^2^
WBC 10^3^/L)	7.35 (1.91) ^1^	9.56 (3.47)	<0.01	7.62 (2.29) ^1^	9.62 (2.51) ^1^	0.01 ^2^	7.16 (1.60) ^1^	9.91 (2.29) ^1^	<0.01 ^2^
PLT (10^3^/L)	227.82 (54.31) ^1^	252.50 (66.30) ^1^	0.24 ^2^	222.35 (60.97) ^1^	256.22 (74.41) ^1^	0.15 ^2^	238.88 (74.27) ^1^	263.72 (62.78) ^1^	0.29 ^2^
ESR (mm/h)	5.00 (5.50)	7.50 (13.50)	0.40	5.00 (11.00)	10.00 (21.80)	0.08	5.00 (6.00)	9.50 (10.50)	0.06
CRP (mg/dL)	0.30 (0.70)	0.43 (0.67)	0.25	0.48 (0.76)	0.78 (7.56)	0.11	0.21 (0.51)	0.34 (0.61)	0.35
B12 (pg/mL)	243.00 (95.00)	276.00 (206.00)	0.33	278.82 (111.03) ^1^	276.00 (229.00)	0.48	331.82 (144.31) ^1^	359.39 (232.18) ^1^	0.67
Folic Acid (ng/mL)	4.40 (3.20)	3.95 (1.80)	0.71	4.00 (4.70)	3.70 (2.10)	0.57	3.80 (13.20)	4.20 (5.40)	0.81

^1^ Normal distribution: mean and standard deviation of the variables were calculated. In all other cases median and interquartile range were calculated. ^2^ *t*-test applied for 2 independent samples. All other tests were performed with the non-parametric Mann-Whitney test. T1; T2; T3, yearly time points 1; 2; 3, respectively. AP1, antipsychotics related to a low to moderate risk of WG, namely aripiprazole, amisulpride, quetiapine, risperidone, and ziprasidone; AP2, antipsychotics related to a higher risk of WG, namely paliperidone, olanzapine, asenapine, clozapine. BMI, body mass index; BP, arterial blood pressure; WBC, white blood cell count; PLTplatelets: ESR, erythrocyte sedimentation rate; CRP, C-reactive protein; HDL, high density lipoprotein; LDL, low density lipoprotein.

**Table 4:** Independence tests for the presence of metabolic syndrome in subgroups of forensic psychiatric patients receiving antipsychotic medication AP1 and AP2, at the three yearly time points (2018, 2019, 2020).

Variables	T1				T2				T3			
	Total	AP1	AP2	*p*-Value	Total	AP1	AP2	*p*-Value	Total	AP1	AP2	*p*-Value
WC (cm)^2^	N/A	N/A	N/A	^-^	N/A	N/A	N/A	^-^	12 (34.3%)	7 (41.2%)	5 (27.8%)	0.40 ^1^
Triglycerides (mg/dL)	8 (22.9%)	3 (17.6%)	5 (27.8%)	0.69	12 (34.3%)	4 (23.5%)	8 (44.4%)	0.19 ^1^	9 (25.7%)	2 (11.8%)	7 (38.9%)	0.12
HDL (mg/dL)	10 (28.6%)	3 (17.6%)	7 (38.9%)	0.26	18 (51.4%)	8 (47.1%)	10 (55.6%)	0.61 ^1^	19 (54.3%)	8 (47.1%)	11 (61.1%)	0.40 ^1^
Systolic BP (mm Hg)	8 (22.9%)	5 (29.4%)	3 (8.6%)	0.44	13 (37.1%)	8 (47.1%)	5 (27.8%)	0.24 ^1^	15 (42.9%)	8 (47.1%)	7 (38.9%)	0.62 ^1^
Diastolic BP (mm Hg)	10 (28.6%)	5 (29.4%)	5 (27.8%)	1.00	11 (31.4%)	6 (35.3%)	5 (27.8%)	0.63 ^1^	12 (34.3%)	9 (52.9%)	3 (16.7%)	0.02 ^1^
Glucose (mg/dL)	6 (17.1%)	3 (17.6%)	3 (16.7%)	1.00	9 (25.7%)	5 (29.4%)	4 (22.2%)	0.71	7 (20%)	3 (17.6%)	4 (22.2%)	1.00
Metabolic Syndrome	10 (28.6%)	5 (29.4%)	5 (27.8%)	1.00	12 (34.3%)	6 (35.3%)	6 (33.3%)	0.90 ^1^	9 (25.7%)	5 (29.4%)	4 (22.2%)	0.71

^1^ Pearson’s independence test was applied. All other tests were performed with the Fisher’s exact test. ^2^ The waist circumference (WC)was measured at T3. AP1, antipsychotics related to a low to moderate risk of WG, namely aripiprazole, amisulpride, quetiapine, risperidone, and ziprasidone; AP2, antipsychotics related to a higher risk of WG, namely paliperidone, olanzapine, asenapine, and clozapine. WC, waist circumference; BP, arterial blood pressure; HDL, high density lipoprotein.

## Data Availability

Data are available upon request to the correspondent authors.
